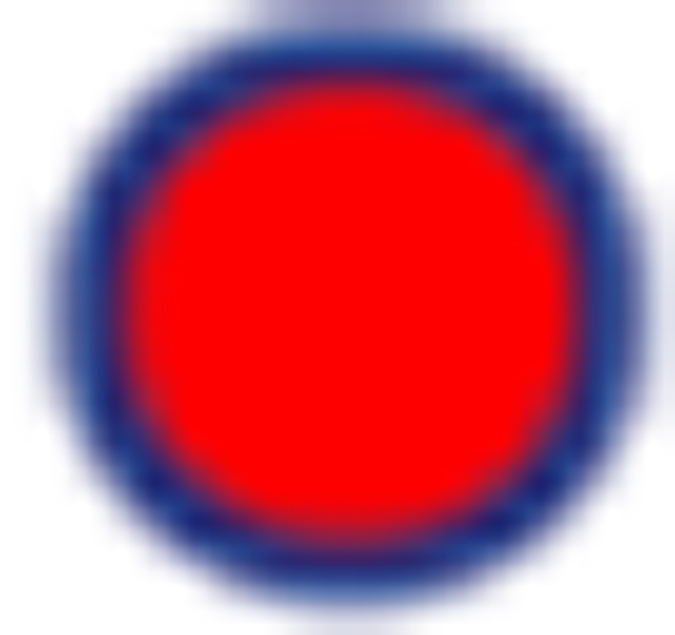# A comparative analysis of high-throughput platforms for validation of a circulating microRNA signature in diabetic retinopathy

**DOI:** 10.1038/srep10375

**Published:** 2015-06-02

**Authors:** Ryan J. Farr, Andrzej S. Januszewski, Mugdha V. Joglekar, Helena Liang, Annie K. McAulley, Alex W. Hewitt, Helen E. Thomas, Tom Loudovaris, Thomas W. H. Kay, Alicia Jenkins, Anandwardhan A. Hardikar

**Affiliations:** 1 Diabetes and Islet biology Group, NHMRC Clinical Trials Centre, Faculty of Medicine, The University of Sydney, Level 6, Medical Foundation Building, 92-94 Parramatta Road, Camperdown, NSW 2050, Australia; 2 Biomarkers Laboratory, NHMRC Clinical Trials Centre, Faculty of Medicine, The University of Sydney, Level 6, Medical Foundation Building, 92-94 Parramatta Road, Camperdown, NSW 2050, Australia; 3 Clinical Genetics Unit, Center for Eye Research Australia (CERA), The University of Melbourne, Royal Victorian Eye and Ear Hospital, Peter Howson Wing, Level 1, 32 Gisborne Street, Melbourne, VIC 3002, Australia; 4 Immunology and Diabetes Unit, St. Vincent’s Institute of Medical Research, 9 Princes St, Fitzroy, VIC 3065, Australia

## Abstract

MicroRNAs are now increasingly recognized as biomarkers of disease progression. Several quantitative real-time PCR (qPCR) platforms have been developed to determine the relative levels of microRNAs in biological fluids. We systematically compared the detection of cellular and circulating microRNA using a standard 96-well platform, a high-content microfluidics platform and two ultra-high content platforms. We used extensive analytical tools to compute inter- and intra-run variability and concordance measured using fidelity scoring, coefficient of variation and cluster analysis. We carried out unprejudiced next generation sequencing to identify a microRNA signature for Diabetic Retinopathy (DR) and systematically assessed the validation of this signature on clinical samples using each of the above four qPCR platforms. The results indicate that sensitivity to measure low copy number microRNAs is inversely related to qPCR reaction volume and that the choice of platform for microRNA biomarker validation should be made based on the abundance of miRNAs of interest.

MicroRNAs (miRNAs/miRs) are 20–22 nucleotide long RNA molecules that are important negative regulators of protein-coding gene expression. They are transcribed by RNAPol-II^1^ to generate mature microRNAs that regulate key processes from embryonic development^2^,^3^ to adulthood^4^,^5^,^6^. MicroRNAs have been intensively studied over the past two decades for their role in regulation of gene expression^7^. However, their importance as biomarkers of disease progression in multiple diseases, including cancer^8^,^9^,^10^,^11^,^12^ and diabetes^13^,^14^,^15^,^16^, is now being identified. MicroRNAs are central to the development and function of the pancreas^17^,^18^,^19^,^20^, as well as glucose-insulin metabolism in target tissues^15^. It is believed that circulating miRNAs may represent a more sensitive and accurate estimation of diabetic retinopathy progression when compared to conventional clinical examination and/or analysis of retinal images.

Currently, quantitative PCR (qPCR) is the favored method for determining miRNA expression, due to its accuracy, simplicity, reproducibility and lower cost than other hybridization or sequencing-based technologies^21^. To validate microRNA biomarkers in clinical diagnosis, microRNAs must be quantified in multiple replicates from a large number of clinical samples to achieve the desired statistical power. Due to this, several manufacturers have developed ultra-high throughput technologies to allow a significantly larger number of assays/samples to be processed and analyzed at a time. Whilst the underlying chemistry of probe-based qPCR assays remains unchanged, advances in microfluidics and nano-engineering have enabled a large number of assays to be carried out in smaller reaction volumes (nanoliters). To date there are no reports systematically comparing microRNA abundance using high throughput technologies with the staple 96-well platform, currently the most accessible platform to the research community. One published platform comparison study evaluated PCR assay efficiencies and platform variability^22^ for two different platforms and a limited number of microRNAs. Although a recent study^23^ has compared multiple qPCR, hybridization and next generation sequencing platforms for cellular microRNA detection, further analysis of qPCR platforms to determine their suitability in reliably measuring low-copy number microRNAs from human biological fluids (plasma/serum) is needed.

Here, we compare four different qPCR platforms from two different vendors, with specific focus on circulating human (plasma) microRNAs. We test the performance of these platforms in validating a microRNA signature for diabetic retinopathy (DR), identified in clinical samples using a discovery approach. We examine both cell and serum RNA isolations on the ViiA7 (Life Technologies), TaqMan Low Density Array (TLDA, Life Technologies, supplemental data), OpenArray (Life Technologies) and Dynamic Array (Fluidigm) systems. The ViiA7 employs the standard 96-well plates (5 μl reaction volume described earlier)^24^ as well as the TLDA cards (1 μl reaction volume). The OpenArray (OA) platform has been described earlier for detection of gene transcripts^25^,^26^ but only recently optimized for miRNA quantification. They utilize unique hydrophobic/hydrophilic interactions to create 33 nl reactions suspended in “through-holes”^26^. The Dynamic Array (DA)^27^ offers the hardware to use any validated TaqMan qPCR assay (mRNA/miRNA/DNA) via microfluidic circuits that create a 15 nl reaction volume. We compared these two high-throughput systems and evaluated each system on their ease of use, relative costs, flexibility of the software and most importantly, the quality of the data obtained, compared with the current “gold standard” 96-well plate platform. These analyses help in identifying qPCR platforms that offer better reproducibility, and present a cogent alternative for researchers interested in profiling multiple miRNAs from a large set of clinical samples. Ultimately, this investigation will guide researchers considering high-throughput miRNA qPCR to make an informed decision.

## Results

### Overview of platforms

**Figure 1:**
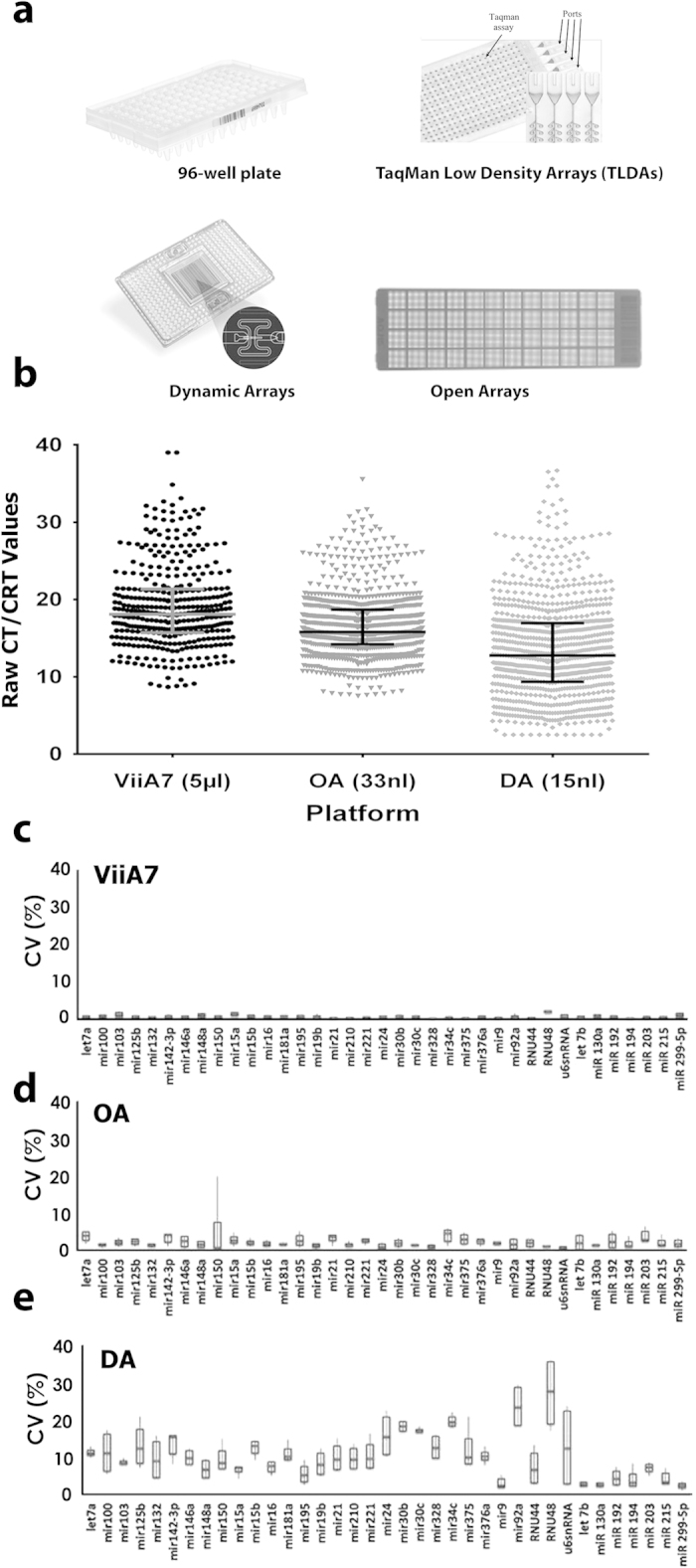
Platform overview and co-efficient of variation analysis. (**a**) Representative pictures of the four platforms. (**b**) CT/CRT values from all samples. Lines indicate median and IQR. Boxplots (median, 10–90th percentile) of the CV distribution for (**c**) ViiA7, (**d**) OA and (**e**) DA.

An overview of the different platforms (Fig. 1a) compared within this study can be found in Table 1. The ease of workflow and cost analysis was computed considering a study size of >100 samples and 48 miRNAs to be screened. The ease of the workflow is also a reflection of the time and experience needed to create the cDNA, pre-amplify it and load it onto the respective platform. All software was supplied by the manufacturer and utilized with default recommended parameters.

The raw cycle threshold (CT) values generated on each platform are plotted in Fig. 1b. A distinct decrease in CT values is seen in the Dynamic Array, most likely due to the increase in the number of pre-amplification cycles and lower dilution factor (1:10 versus 1:40 for the OpenArray), as recommended for the DA platform. The median CT value for the entire miRNA dataset analyzed is 18.09 for the ViiA7, 15.79 for OA and 12.76 for DA.

### Platform variability

To assess the variability of each platform, the co-efficient of variation (CV) was calculated for each microRNA assay (Fig. 1c–e and Supplementary Fig. 1). The median CV of the replicates was lowest in the 5 μl 96-well platform (0.6%), ranging from 0.1 to 1.9% (Fig. 1c), then OA at 2.1% (range 0.7–4.6%; Fig. 1d), TLDA at 8.3% (range 0.3–19.1%; Supplementary Fig. 1) and DA at 9.5% (range 2.2–27.6%; Fig. 1e).

**Figure 2:**
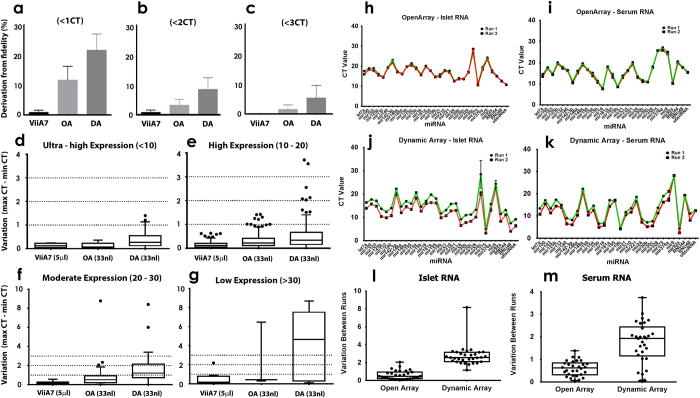
Platform variability and reproducibility. (**a**–**c**) Fidelity scoring using (**a**) 1 CT/CRT, (**b**) 2 CT/CRT or (**c**) 3 CT/CRT cut-off. Data is presented as deviation from fidelity (100%) and plotted as mean + SEM. (**d**–**g**) Tukey boxplots (median + IQR) of variation between replicates (maximum CT/CRT—the minimum CT/CRT) for the four expression levels, (**d**) ultra-high (CT/CRT <10), (**e**) high (CT/CRT 10-20), (**f**) moderate (CT/CRT 20-30) and (**g**) low (CT/CRT >30). Dotted line is 1, 2 or 3 CT/CRT cut-off used in fidelity scoring. (**h**–**m**) RNA from islet cells and human serum was run twice on the (**h**,**i**) OA and (**j**,**k**) DA (same batch/lot). Data are mean ± SD. The average difference (boxplots, median + min/max) between runs for each miRNA tested for cellular miRNAs is plotted in (l) and for serum miRNAs in (**m**).

As an indicator of overall reproducibility, the fidelity of each platform was assessed. Fidelity was calculated as the percentage of replicates that differed by less than 1, 2 or 3 CT values and is presented as deviation from fidelity in Fig. 2a–c and Supplementary Fig. 2. The standard 96-well format attained 99.23% fidelity for both 1 and 2 CT values, and 100% fidelity for the 3 CT cut-off (Fig. 2a–c). Both OpenArray and Dynamic Array platforms performed capably (Fig. 2a–c); 88.1% (OA) and 77.78% (DA) of replicates differed by less than 1 CT, 96.29% (OA) and 91.27% (DA) less than 2 CTs, and 98.41% (OA) and 94.44% (DA) less than 3 CTs. Neither of the high-throughput technologies were able to attain 100% fidelity using these CT cut-off points. This variation of the CT values would effectively translate to ~8-fold difference in transcript abundance. It is worth noting that even if an 8-fold variation between replicates is considered acceptable, 1.59% (OA) and 5.56% (DA) of replicates may still show greater than 8-fold variability.

CT values were then segregated into expression levels and the replicate variation plotted (Fig. 2d–g and Supplementary Fig. 3). Ultra-high expression was considered to be CT values less than 10, high expression defined to be a CT value between 10.01 and 20.00, moderate expression between 20.01 and 30.00, and low expression above 30.01. Replicate variability increased across all platforms as the transcript abundance decreased. This was especially apparent in the high-throughput platforms, where replicate variability increased substantially when the transcript was present at moderate or low expression (Fig. 2d–g and Supplementary Fig. 3). The 96-well plate platform (ViiA7) maintained a CT variation of less than 1 cycle until the target transcript was expressed at low levels (>30.01 CT; Fig. 2g). The ViiA7 platform thus truly represents the “gold standard” amongst these qPCR platforms as any variation seen for such low level transcripts is potentially due to the Poisson distribution^28^. When dealing with low copy transcripts, the Poisson distribution predicts that in a large number of replicates containing an average of one copy of starting template per reaction (rxn), approximately 37% rxns should actually have no copies, only 37% rxns should contain one copy, and 18% rxns should contain two copies^28^. Therefore, reliable detection of single copy transcripts in any sample calls for a large number of replicates to assess statistical significance and overcome the limitations of Poisson distribution.

### Reproducibility

To determine how reproducible the high-throughput technologies are, RNA extracted from human islet cells and serum was run twice on each platform by two different users (RJF and MVJ). The average difference between the two sets of replicates is plotted in Fig. 2h–m. The OpenArray system produced fairly reproducible results, with the greatest variation being 2.06 cycles, whilst the Dynamic Array system demonstrated larger variation (up to 8.17 cycles; Fig. 2j–m). Interestingly, the second repeat on the Dynamic Array system produced consistently lower CT values. All aspects of the procedure were kept constant so we cannot comment on why this may have occurred.

### miRNA signature detection

**Figure 3:**
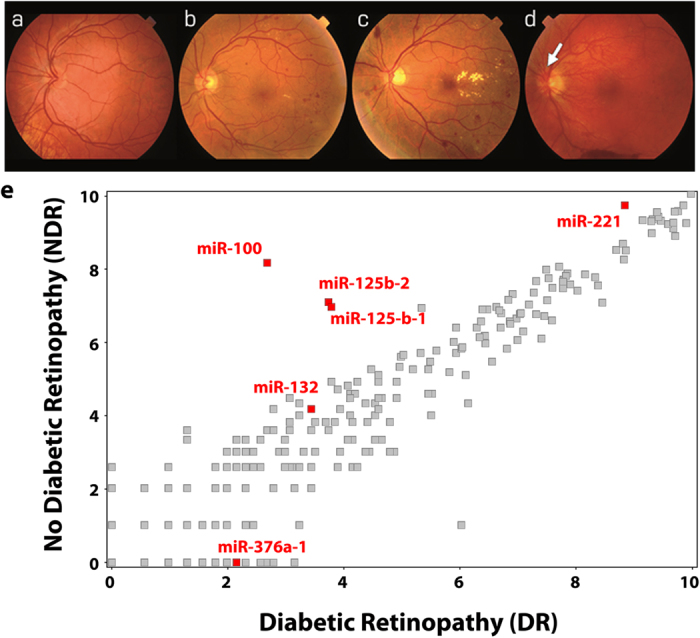
A circulating microRNA signature for Diabetic Retinopathy (DR). Fundus photographs showing the clinical spectrum of DR: (**a**) a normal retina—no DR; (**b**) mild non-proliferative DR, with hemorrhages, microaneurysms and hard exudates; (**c**) non-proliferative DR; (**d**) proliferative DR, at the optic disc (white arrow) and pre-retinal hemorrhage in the inferior retina. (**e**) MicroRNAs consistently identified in plasma of age and gender matched diabetic individuals with or without DR following analysis of smallRNA sequencing (see text). The miRs that constitute part of the DR biomarker signature are displayed in red.

**Figure 4:**
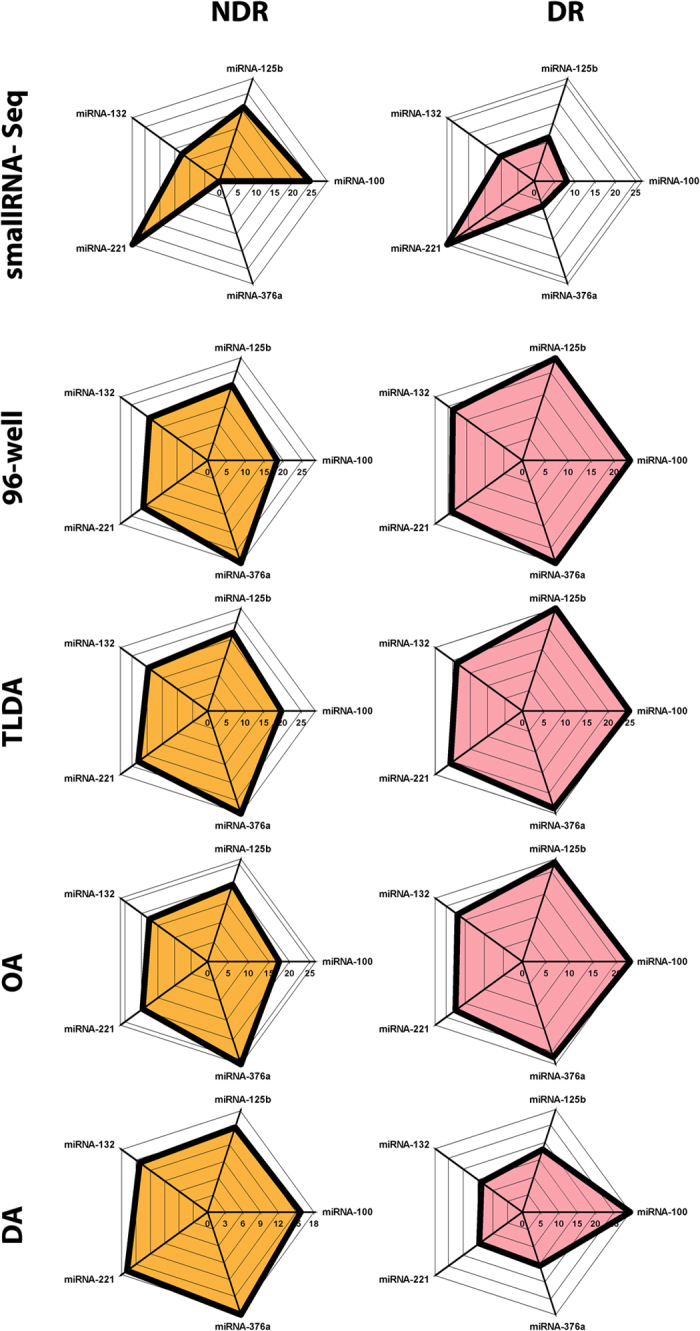
NDR and DR biomarker signature profiles. Radar plots of miRNAs signature profiles from smallRNA sequencing of two age and gender matched subjects (with (DR) and without (NDR) DR). Based on DR/NDR difference 5 miRNAs were chosen as a “DR biomarker signature”. Radar plots were generated using the CT/CRTs for these miRNAs on the four platforms. The results show the profile of the miRNA signature plot differs between NDR and DR in all platforms. The DA delivers a different miRNA signature profiles for DR and NDR. Scale is normalized reads for smallRNA-Seq and CT/CRT for 96-well, TLDA, OA and DA.

One of the ultimate goals of most miRNA expression studies is to determine a biomarker signature related to particular pathology. To assess a biomarker signature for diabetic retinopathy (DR), we used unbiased small RNA sequencing of plasma samples from diabetic individuals with or without retinopathy. While analyzing samples from subjects with (DR) and without (No DR; NDR) diabetic retinopathy (Fig. 3a–d) we selected five miRNAs based on consistent differences between DR and NDR subjects and assessed the detection of such a “fingerprint” (Fig. 3e) using the four different qPCR platforms. We carried out rigorous analyses (Fig. 4) to compare differences in expression of this microRNA signature amongst DR and NDR subjects. Interestingly, these analyses demonstrate that the features of miRNA expression (denoted by shaded areas in Fig. 4) retain their profile across three qPCR platforms - ViiA7 (96-well), TLDA and OA platforms - whilst these same features appear skewed for the DA platform. As expected, the area and feature properties are different for the sequencing platform and vary between the DR and NDR groups amongst the sequencing profile.

### Correlation between platforms and cluster analysis

**Figure 5:**
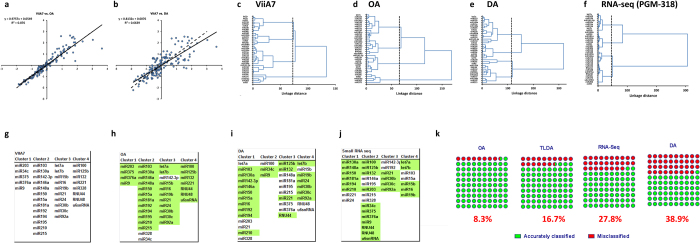
Platform concordance and cluster analysis (**a**–**b**) Correlation of Z-scored transformed results between ViiA7 and either (**a**) OA or (**b**) DA. The hypothetical trend line of slope = 1 (dashed line) represents complete concordance, with the actual trend plotted as a solid line. The slope of the trend line (upper left corner) shows the deviation from 1. (**c**–**f**) Dendrograms of cluster analysis, (**c**) ViiA7, (**d**) OA, (**e**) DA, and (**f**) small RNA-Seq. Dotted line represents the cut-off value for the number of clusters (as determined by the Mojena rule). (**g**–**j**) MicroRNA cluster classification. miRNA clusters as classified by the (**g**) ViiA7, (**h**) OA, (**i**) DA, and (**j**) small RNA-Seq. Highlighted miRNAs represent members belonging to the same cluster as those in the ViiA7 cluster analysis. (**k**) Percentage dot plot of miRNA cluster classification. 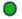 indicates the percentage of miRNAs that have been correctly classified as compared to the cluster analysis from the ViiA7 platform, while 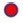 indicates those that are misclassified. 8.33% of microRNAs were assigned to different clusters by OA, 16.66% by TLDA, 27.77% by small RNA-Seq, and 38.88% by DA.

To determine intra-platform consistency, the correlation between platforms was analyzed. We assessed 42 miRNAs (including the DR signature miRs) in four human subject samples (two islet cell samples and two serum samples; a total of 168 matched measurement pairs) and plotted these as pair-wise comparisons against ViiA7 (“gold standard”) platform for OA, DA (Fig. 5a,b) and TLDA (Supplementary Fig. 4). All platforms show good correlations with ViiA7, with the R^2^ values 0.88 and 0.66 for OA and DA respectively. The TLDA platform had an R^2^ value of 0.91 (Supplementary Fig. 4). The lowest compression was observed in OA platform (slope: 0.98) and TLDA (slope: 0.94) whereas DA platform showed the moderate compression with slope 0.81.

Cluster analysis was implemented to determine the ability of these qPCR platforms in allocating miRNAs into distinct clusters. Each platform grouped the microRNAs into four distinct clusters (Fig. 5c–f and Supplementary Fig. 5). Again, the 96-well ViiA7 platform was used as the benchmark; clusters produced by the other platforms were directly compared to this “gold standard” (Fig. 5g–j and Supplementary Fig. 6). Comparison of all of the platforms (qPCR and small RNA-Seq) reveal that approximately 8% of miRNAs were assigned to different clusters by OA, 17% by TLDA, 28% by small RNA-Seq, and 39% by DA (Fig. 5k).

## Discussion

The appeal of high-throughput technologies is readily apparent; they provide a significant decrease in sample processing time and reagents consumed, with a substantial increase in the volume of data generated in the same time. For researchers within the burgeoning field of miRNA biomarker research, such a tool is immensely desirable, especially when dealing with a multitude of clinical samples from large trials. Such assay performance would also be required should a miRNA profile related to diabetes, reach clinical practice. It is imperative that technologies used for quantitative research or in clinical practice be accurate, sensitive and reproducible, and high-throughput platforms must present a similar (if not greater) level of reproducibility offered by current high sensitivity/low-throughput platforms.

The two high-throughput technologies discussed here represent the two most contemporary high-throughput qPCR platforms. The Dynamic Array platform offers a finely crafted plate with a set of individually pressure-tested valves that control the flow of reagents into the microfluidic plate. The OpenArray platform provides a high density PCR plate/slide that allows automated sample loading using a specially designed robotic device (Accufill™). The major difference is that the OpenArray comes with pre-printed miRNA assays while the Dynamic Array provides flexibility to choose the miRNA assays just before setting up the plate. The DA platform thus provides only the “hardware” for users to perform qPCR. Although this flexibility is an advantage, the limitation of the DA is that it heavily depends on the compatibility of the pooled RT primers with the TaqMan real-time PCR assays. Of the 48 miRNAs selected from the TaqMan primer pool A (Life Technologies, CA) six of these assays could not be read on the DA due to incompatibility between the RT and PCR primers. Since the proprietary primer pools are compatible with the primers printed in TaqMan Low Density Arrays (TLDAs) or OpenArray plates only, the individual assays available from Life Technologies may not be compatible with the multiplex RT pools. Of course, users can avoid this by ordering customized primer pools from Life Technologies to assess a specific set of miRNAs.

In our analysis, the OpenArray system was the most reproducible high-throughput platform tested, with less inter- and intra-run variation than the Dynamic Array. This conclusion correlates with a recent study comparing these platforms for screening genomic mutations in the Ashkenazi Jewish community^25^. The OA platform also proffers a simple, cost-effective workflow, with user-friendly software for further analyses.

Our data demonstrates that replicate variability was exacerbated when quantifying low abundance transcripts on any of the platforms. As shown in Fig. 2d–g, this variability is especially apparent in the high-throughput platforms, highlighting a limitation of these systems. Screening low abundance transcripts on these platforms should be avoided, as even the implementation of a pre-amplification step was insufficient to reduce CT variability. Increasing the number of pre-amplification cycles is not recommended as it would amplify any bias that is introduced in the process. Users of Dynamic Arrays could expect 5.56% of their assays to demonstrate more than 8-fold variability. Any data obtained using DAs with differences less than or around 8-fold need to be re-validated using a high sensitive PCR technology, such as the 96-well platform described herein. For miRNAs detected around a CT value of 30, the standard qPCR format proves more reliable.

For medium-throughput studies, researchers can utilize the TLDA platform. We found a comparable level of reproducibility on this platform when using cards from the same lot (Supplementary Fig. 1), but have observed considerable lot-to-lot variation^29^.

Correlation between technologies is required to translate relative gene expression data on different qPCR platforms. Since CT values cannot be directly compared across platforms due to the differences in sample preparation, reaction volume and detection technologies, correlation between z-scored transformed data (Fig. 5a,b) is a reliable method to compare the data obtained from multiple qPCR technologies. All of the technologies assessed correlated well with the standard 96-well platform, however the OpenArray platform deviated the least from the theoretical ideal value of 1, demonstrating that it correlates extremely well with the staple 96-well format. As such, up or down scaling miRNA qPCR reactions between the ViiA7 and OA platforms will produce parallel data.

Segregation of miRNAs based upon their expression profile is useful for biomarker identification. Our cluster analysis (Fig. 5c–k) demonstrated that data obtained from all platforms were able to create four distinct miRNA clusters; however, the members of these clusters differed between the platforms. The Dynamic Array generated fundamentally different clusters, making it difficult to identify our test DR miRNA signature (generated through smallRNA sequencing). The OpenArray platform generated the most similar clusters (compared to 96-well), with similar segregation of miRNA DR signature members into clusters giving confidence in validating disease-related microRNA signatures. Although quantitative real time PCR platforms have different sensitivities and efficacies for reproducing the miRNA DR signature we assessed the similarity in data obtained from qPCR versus our sequencing platform. Interestingly, the small RNA-Seq data also generated four distinct clusters, with 27% of the miRNAs misassigned to the four clusters. This may be due to the fact that qPCR and next generation sequencing employ vastly different chemistries to quantitate miRNAs and that the outcome of such comparisons would be largely impacted by the depth of sequencing achieved.

The miRNA signature profile generated by our smallRNA sequencing, although representative, demonstrates the importance of rigorously analyzing new technologies, especially those that may become routine for biomarker discovery and validation and potentially be used in clinical practice. Figure 4 demonstrates the differences in miRNA signature profile detected by multiple platforms.

This report demonstrates the importance of multiple technical factors that can influence the conclusions derived from miRNA qPCR data. Although there are differences in the respective detection systems (QuantStudio for OpenArrays, Biomark HD for Dynamic Arrays), the size of the reaction may also affect the reproducibility of these high-throughput systems. Although both platforms have a reaction volume in nanoliters, the OpenArray reaction volume is 2.2-fold more than the Dynamic Arrays. During the optimization of our standard (96-well) qPCR analysis for detection of low-copy miRNA/mRNA transcripts, the amplification curves differed by 0.5 cycles when the reaction volume was reduced from 10 μl to 5 μl(data not shown). Considering the Poisson distribution of miRNA templates, this difference can be significant when more than two-fold difference is factored in, even at the nanoliter range. Although each of the platforms discussed have their own advantages/disadvantages, users need to consider all aspects before choosing an analytical platform, and ideally should test their data across another high-sensitivity platform such as the 96-well platform. These observations provide a useful guide for researchers planning to switch to high-throughput qPCR technologies for quantitative assessment of gene transcripts in large number of research/clinical trial samples. Furthermore, the development of a sensitive, reliable and minimally invasive test with high predictive power for clinical stratification of DR will provide a surrogate marker for clinical trials, improve prognosis information and guide development of future therapies aimed towards preventing the vascular complications of diabetes.

## Methods

### Samples

The study was approved by the University of Sydney Ethics Committee and all samples were collected following written informed consent. Total RNA was isolated from human islets (Islet Transplant Consortium) and from serum samples, collected from individuals with or without diabetes. The study was carried out in accordance with the approved guidelines. Samples were tested for a total of 48 miRNAs, selected based upon their expression in small RNA sequencing, their relevance to islet biology or diabetes, or their suitability as housekeeping transcripts. However, six of these 48 miRNAs were identified to be incompatible on the Fluidigm system (see discussion) and therefore all of the analysis for the two platforms is based on comparison of the expression of these 42 miRNAs on all the platforms tested. The 42 miRNAs include three “housekeeping” miRNAs (U6, RNU44 and RNU48). Each PCR reaction was undertaken at least in duplicate. Technical replicates were also included within the same run as well as in different runs, and repeated by two or three users (RJF, MVJ and AAH). No significant variation was seen in data obtained by the three users.

### ViiA7

To aid comparison with the pre-amplified samples run on the high-throughput technologies, samples were pre-amplified and run in the 96-well format using 5 μl reaction volumes. Reverse transcription (RT) and pre-amplification (PA) was completed using the Megaplex RT/PA Primer Pools (Life Technologies) using the manufacturer’s protocol, with minor changes. Briefly, each sample had an RNA input of 100 ng (measured by Nanodrop), went through 12 cycles of PA and was diluted 1:40 in nuclease-free water. qPCR was undertaken in 0.1 ml optically clear 96-well plates using TaqMan MicroRNA Primer/Probe mixes and TaqMan Fast Universal PCR Master Mix (2X), No AmpErase UNG (Life Technologies).

### OpenArray

RT and PA were undertaken with Megaplex RT/PA Primer Pools using the manufacturer’s OpenArray microRNA Panel protocol as described elsewhere^29^. Briefly, each sample (100 ng input), underwent 12 PA cycles, then was diluted 1:40 in 0.1X TE (pH 8.0), combined with TaqMan OpenArray PCR Master Mix and loaded onto TaqMan OpenArray Human MicroRNA Panel (AccuFill™ system). qPCR was completed using the QuantStudio 12K Flex (Life Technologies).

### Dynamic Array

RT and PA were undertaken with Megaplex RT/PA Primer Pools using the protocol supplied by Fluidigm Corporation. Each sample (input 100 ng), underwent 16 PA cycles (recommended by the manufacturer), then was diluted 1:10 in nuclease-free water, combined with TaqMan MicroRNA Primer/Probe mixes and TaqMan Fast Universal PCR Master Mix (2X), No AmpErase UNG, and loaded onto a 48 × 48 Dynamic Array IFC (Integrated Fluidic Circuit) using the IFC Controller MX. qPCR was performed on the BioMark system (Fluidigm Corporation).

### Next generation sequencing

RNA was isolated from plasma samples using protocol described earlier^30^. SmallRNA libraries generated using the Ion Total RNA-Seq Kit v2 were processed through emulsion PCR using OneTouch 2 (Life Technologies) and the Ion PGM™ Template OT2-200 Kit, then enriched using the OneTouch ES (all Life Technologies). One sample was loaded per Ion-318^™^ chip and sequenced on Ion PGM using the Ion PGM™ Sequencing 200 Kit v2 (all Life Technologies). The aligned BAM files were analyzed using Strand NGS (Strand Life Sciences) and differential miRNA expression was determined by a two-fold change in normalized read number.

### Statistical analysis

The data were analyzed using Statistica for Windows analytical software (ver. 12.0, StatSoft, Tulsa, OK). For intra-platform reproducibility analysis the coefficient of variation (CV%) was calculated between replicate measurements. Data was obtained from the multiple measurements (lowest number of replicates = 2, highest = 8) of 40 transcripts in four samples. miR-140 and miR-320 were excluded due to the lack of results on TLDA and DA platforms respectively.

CT/CRT values could not be compared directly so they were transformed into Z-scores within each platform. Pearson correlation and linear fits were calculated. Level of compression or expansion of data measured on the different platforms were evaluated by comparing the slope of the best fitted line of a least square linear regression of the Z-scores between pairs to the “ideal” slope of 1.

Ward’s linkage method and Euclidean distances were used for cluster analysis. Separate dendrograms were generated for each analytical platform. Number of clusters was based on Mojena rule or distances between the dendrogram knots. In each analytical method four clusters were observed. The miRNAs of each cluster were then calculated using k-means method. MicroRNAs with >15% missing data (across all platforms) were excluded from the calculations; otherwise missing data were replaced by mean. Clusters concordance was estimated by comparing the clusters generated by other platforms with the ViiA7 (“gold standard”). The assignment was declared in concordance if at least two miRNAs were placed in the same cluster by different methods.

## Additional Information

**How to cite this article**: Farr, R. J. *et al.* A comparative analysis of high-throughput platforms for validation of a circulating microRNA signature in diabetic retinopathy. *Sci. Rep.* **5**, 10375; doi: 10.1038/srep10375 (2015).

## Supplementary Material

Supplementary Information

## Figures and Tables

**Table 1:** Overview of platforms compared in this study.

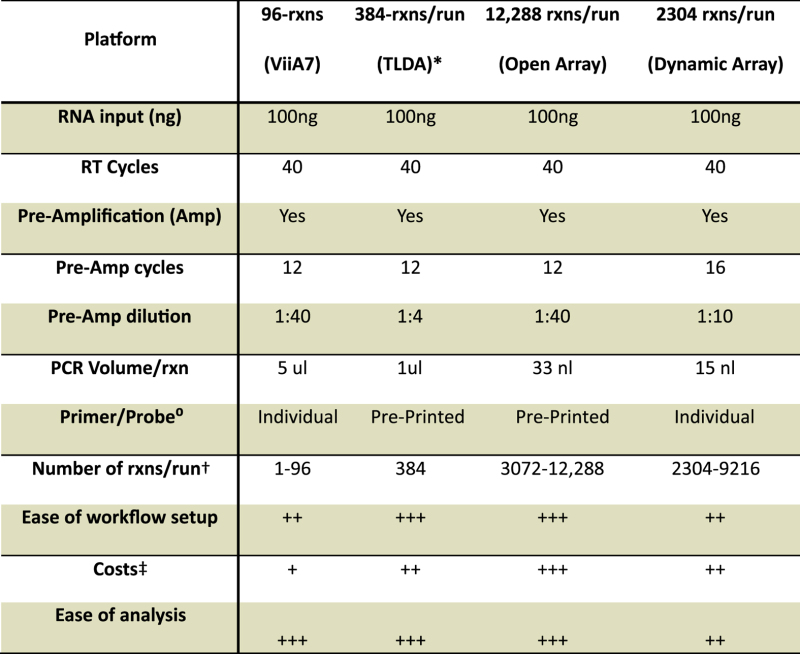

^*^See supplemental data.

^°^The use of individually available TaqMan primer/probe assays or pre-printed TaqMan primer/probe assays. Note: we recommend using the individual TaqMan assays for the panel of microRNAs proposed as there may be compatability issues between off-the-shelf megaplex primer pools and the individual microRNA TaqMan assay.

^†^Based on the possible reactions that can be assessed on each platform in a single run on a single instrument.

^‡^Based on actual total costs involved in Sydney, Australia.

^§^Based on our analysis of software capabilities, robustness and ease of use of the software supplied by the manufacturer and ranked by 3 independent users.

**Figure:**
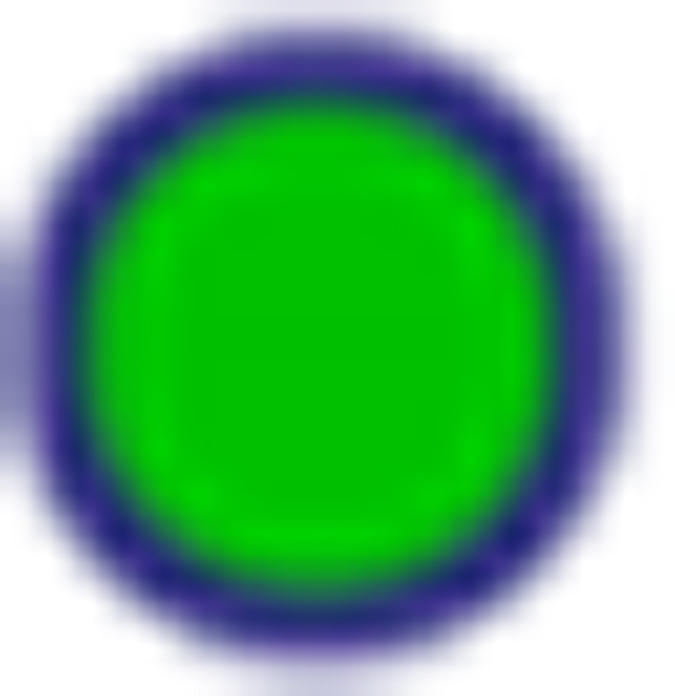


**Figure:**