# Supplementary material for: A comparative analysis of high-throughput platforms for validation of a circulating microRNA signature in diabetic retinopathy
Source: Sci Rep. 2015 Jun 2;5:10375. doi: 10.1038/srep10375 (PMC4649912; doi:10.1038/srep10375)
Supplement: Supplementary Information [file srep10375-s1.doc]

**Online Supplementary Data**

**A comparative analysis of high-throughput platforms for validation of a circulating microRNA signature in diabetic retinopathy**

Ryan J. Farr^1^, Andrzej S. Januszewski^2^, Mugdha V. Joglekar^1^, Helena Liang^3^, Annie K McAulley^3^, Alex W Hewitt^3^, Helen E. Thomas^4^, Tom Loudovaris^4^, Thomas W.H. Kay^4^, Alicia Jenkins^2^ and Anandwardhan A. Hardikar^1,5^.

1. Diabetes and Islet biology Group, NHMRC Clinical Trials Centre, Faculty of Medicine, The University of Sydney, Level 6, Medical Foundation Building, 92-94 Parramatta Road, Camperdown, NSW 2050, Australia.
2. Biomarkers Laboratory, NHMRC Clinical Trials Centre, Faculty of Medicine, The University of Sydney, Level 6, Medical Foundation Building, 92-94 Parramatta Road, Camperdown, NSW 2050, Australia.
3. Clinical Genetics Unit, Center for Eye Research Australia (CERA), The University of Melbourne, Royal Victorian Eye and Ear Hospital, Peter Howson Wing, Level 1, 32 Gisborne Street, Melbourne, VIC 3002, Australia
4. Immunology and Diabetes Unit, St. Vincent’s Institute of Medical Research, 9 Princes St, Fitzroy, VIC 3065, Australia

^5.^Address all correspondence to:

Anandwardhan A. Hardikar, PhD

Diabetes and Islet Biology Group, NHMRC Clinical Trials Centre,

Faculty of Medicine, The University of Sydney,

Level 6, Medical Foundation Building,

92-94 Parramatta Road, Camperdown,

NSW 2050, AUSTRALIA

Phone: +61 2 9562 5071 | Fax: +61 2 9565 1863

E-mail: anand.hardikar@ctc.usyd.edu.au

Web: <http://www.isletbiology.com/> | http://www.ctc.usyd.edu.au/

**TLDA Method**

RT and pre-amp were undertaken with Megaplex RT/PA Primer Pools using the manufacturer’s protocol. Each sample had 80-100 ng RNA input (as measured by Nanodrop), 12 cycles of pre-amplification and was diluted 1:4 with 0.1 x TE pH 8.0. qPCR was completed using TaqMan® Low Density Array human microRNA Panel following the manufacturer’s protocol.


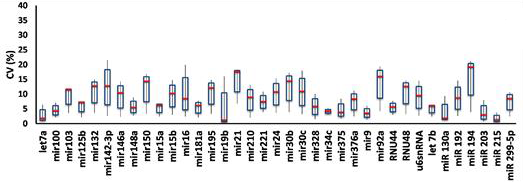


**Supplementary Figure 1 | TLDA coefficient of variation analysis.** Box and whiskers plot of the CV distribution for TLDA. Boxes indicate median with upper and lower quartile boundaries, whiskers are 10-90^th^ percentile.


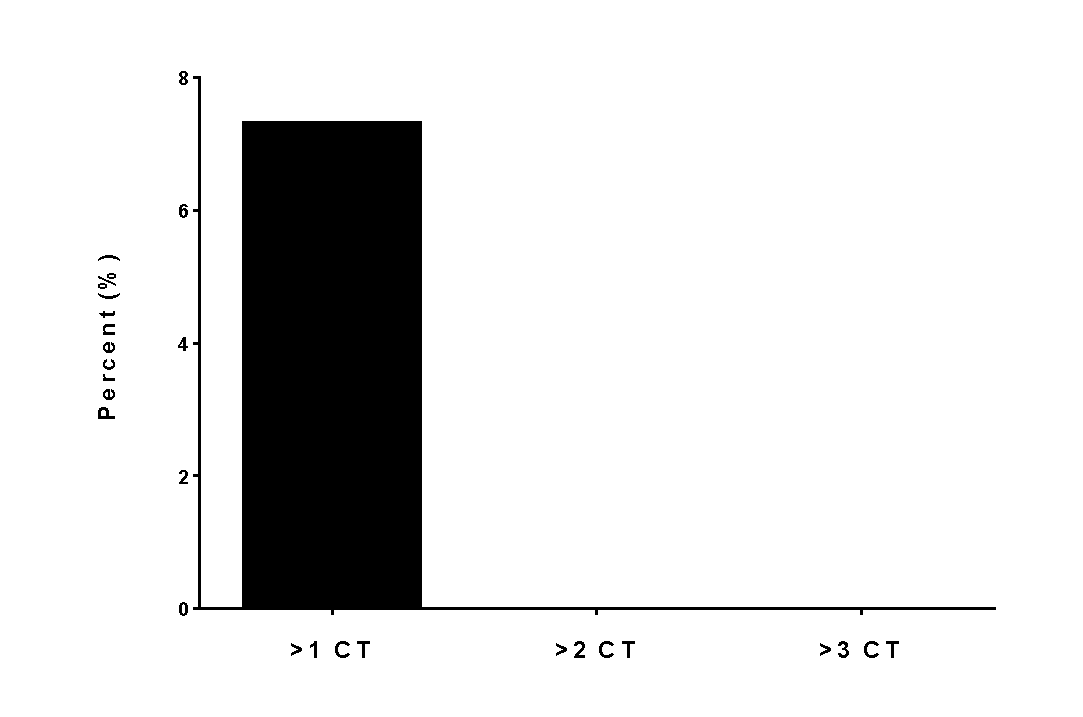


**Supplementary Figure 2 | Fidelity scoring of the TLDA platform.** Fidelity scoring is based upon the percentage of replicates for each platform that differed by less than (A) 1 CT, (B) 2 CT or (C) 3 CT value. Data is presented as deviation from fidelity (100%).


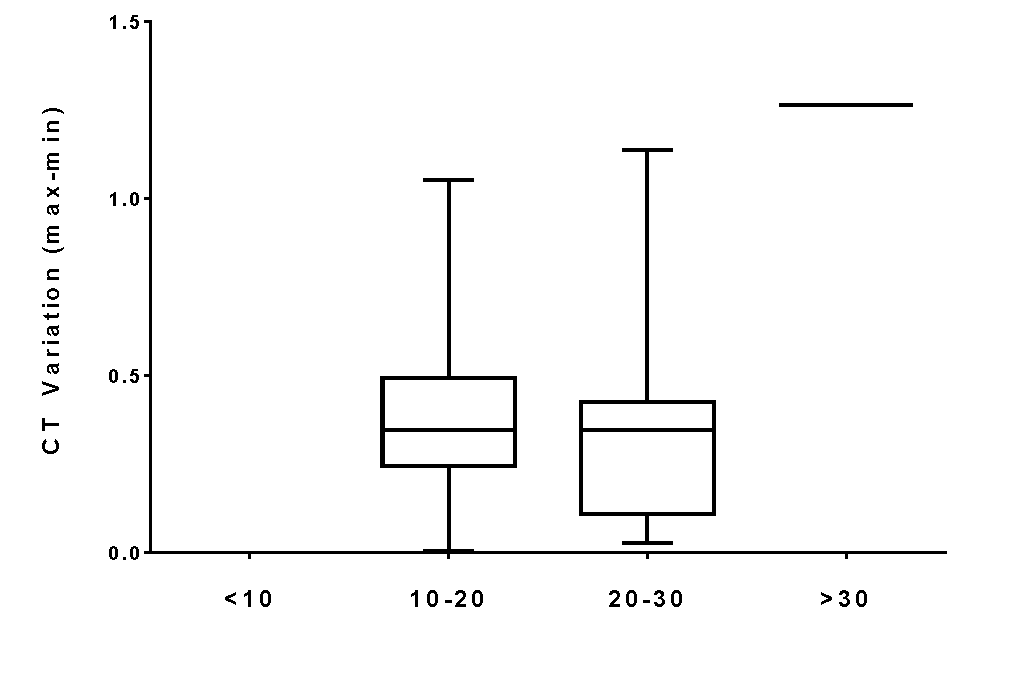


**Supplementary Figure 3 | Replicate Variation on the TLDA Platform.** Spread of variation between replicates (measured as the maximum CT – the minimum CT) for the four expression levels, ultra-high (CT<10), high (CT 10-20), moderate (CT 20-30) and low (CT>30).


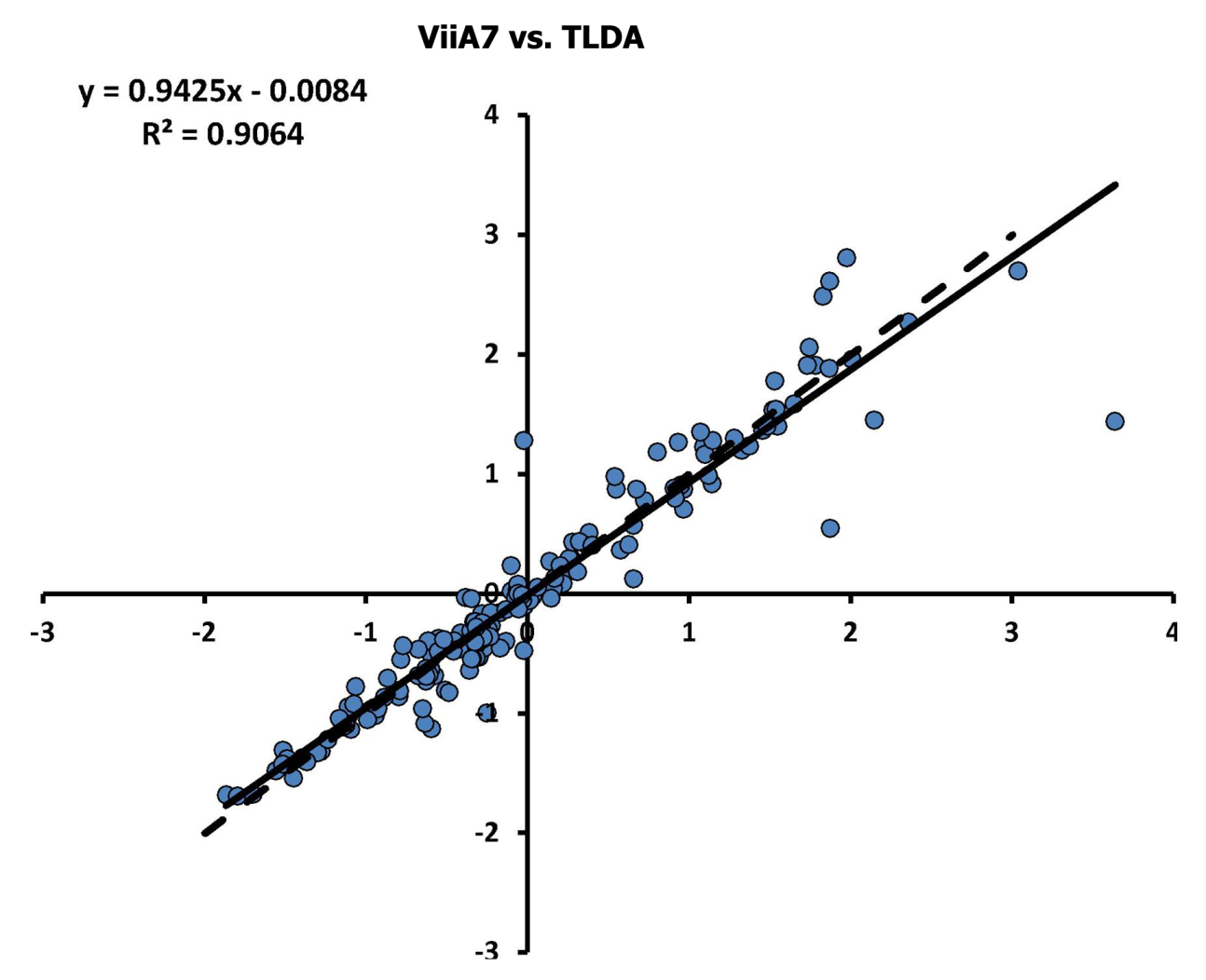


**Supplementary Figure 4 | Correlation of TLDA with ViiA7.** Correlation of Z-scored transformed results between ViiA7 and TLDA. The hypothetical trend line of slope = 1 (dashed line) and the actual trend (solid line) between measurements obtained from two platforms is plotted. The slope of the trend line (upper left corner) is showing the deviation from 1.


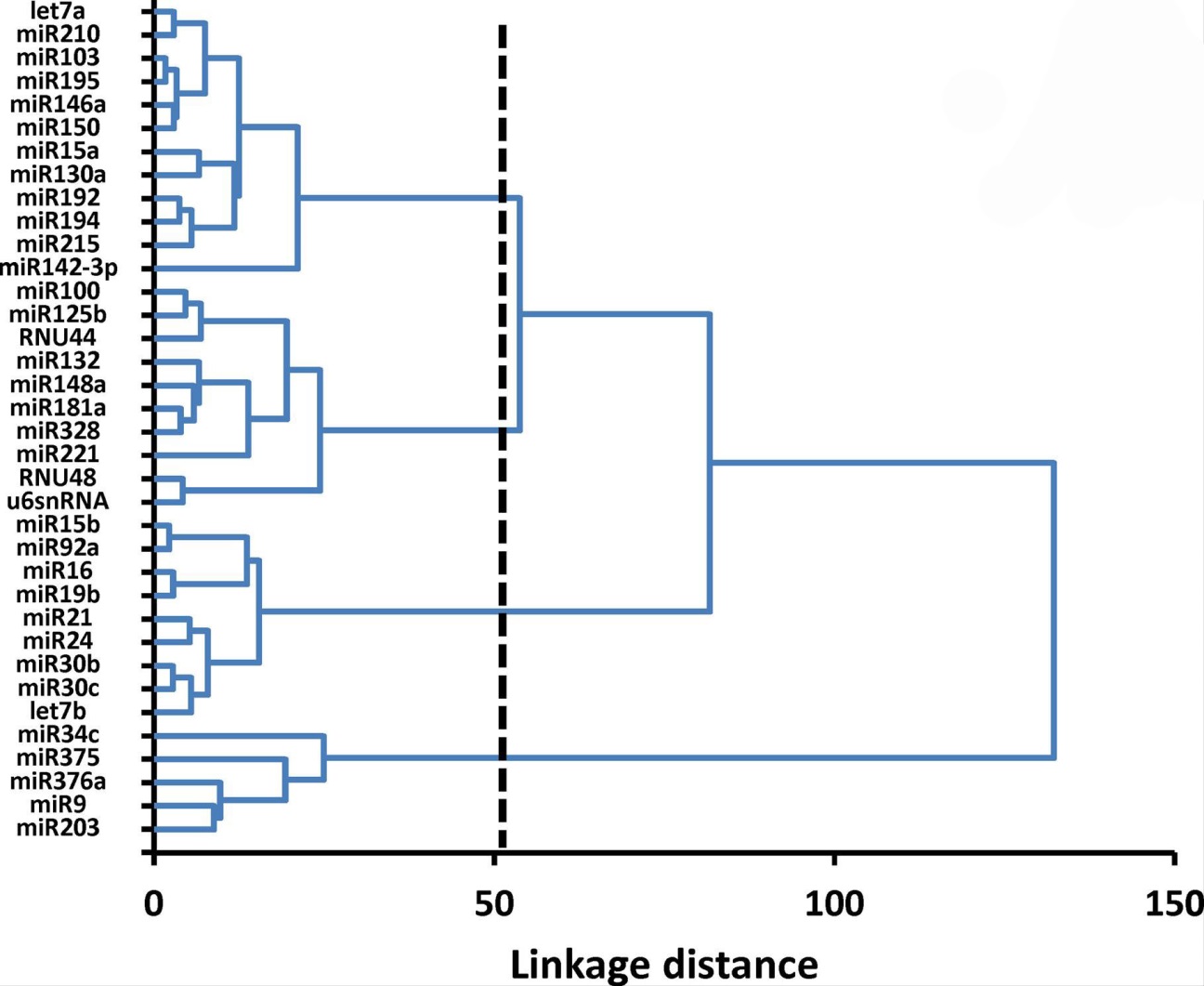


**Supplementary Figure 5 | miRNA cluster analysis by TLDA.** Dendrograms of cluster analysis from TLDA. Dotted line represents the cut-off value for the number of clusters determination (using the Mojena rule).


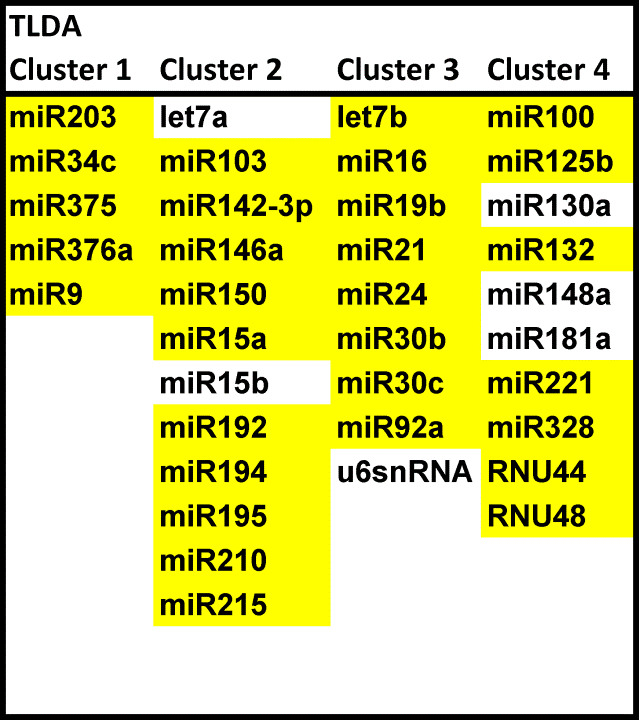


**Supplementary Figure 6 | Comparison of microRNA classification into clusters by TLDA.** Highlighted miRNAs have been allocated to the same cluster (as ViiA7) by TLDA. 16.67% of microRNAs were assigned to different clusters by the TLDA platform.
